# A protocol for annotation of total body photography for machine learning to analyze skin phenotype and lesion classification

**DOI:** 10.3389/fmed.2024.1380984

**Published:** 2024-04-09

**Authors:** Clare A. Primiero, Brigid Betz-Stablein, Nathan Ascott, Brian D’Alessandro, Seraphin Gaborit, Paul Fricker, Abigail Goldsteen, Sandra González-Villà, Katie Lee, Sana Nazari, Hang Nguyen, Valsamis Ntouskos, Frederik Pahde, Balázs E. Pataki, Josep Quintana, Susana Puig, Gisele G. Rezze, Rafael Garcia, H. Peter Soyer, Josep Malvehy

**Affiliations:** ^1^Dermatology Department, Hospital Clinic and Fundació Clínic per la Recerca Biomèdica—IDIBAPS, Barcelona, Spain; ^2^Frazer Institute, The University of Queensland, Dermatology Research Centre, Brisbane, QLD, Australia; ^3^V7, London, United Kingdom; ^4^Canfield Scientific Inc., Parsippany-Troy Hills, NJ, United States; ^5^ISAHIT, Paris, France; ^6^Torus Actions & Belle.ai, Ramonville-Saint-Agne, France; ^7^IBM Research, Haifa, Israel; ^8^Coronis Computing SL, Girona, Spain; ^9^Computer Vision and Robotics Group, University of Girona, Girona, Spain; ^10^Remote Sensing Lab, National Technical University of Athens, Athens, Greece; ^11^Fraunhofer Heinrich-Hertz Institute, Berlin, Germany; ^12^HUN-REN Institute for Computer Science and Control, Budapest, Hungary; ^13^Medicine Department, University of Barcelona, Barcelona, Spain; ^14^CIBER de Enfermedades raras, Instituto de Salud Carlos III, Barcelona, Spain; ^15^Dermatology Department, Princess Alexandra Hospital, Brisbane, QLD, Australia

**Keywords:** artificial intelligence, total body photography, dermatology, melanoma, computer—aided diagnosis, computer image analyses

## Abstract

**Introduction:**

Artificial Intelligence (AI) has proven effective in classifying skin cancers using dermoscopy images. In experimental settings, algorithms have outperformed expert dermatologists in classifying melanoma and keratinocyte cancers. However, clinical application is limited when algorithms are presented with ‘untrained’ or out-of-distribution lesion categories, often misclassifying benign lesions as malignant, or misclassifying malignant lesions as benign. Another limitation often raised is the lack of clinical context (e.g., medical history) used as input for the AI decision process. The increasing use of Total Body Photography (TBP) in clinical examinations presents new opportunities for AI to perform holistic analysis of the whole patient, rather than a single lesion. Currently there is a lack of existing literature or standards for image annotation of TBP, or on preserving patient privacy during the machine learning process.

**Methods:**

This protocol describes the methods for the acquisition of patient data, including TBP, medical history, and genetic risk factors, to create a comprehensive dataset for machine learning. 500 patients of various risk profiles will be recruited from two clinical sites (Australia and Spain), to undergo temporal total body imaging, complete surveys on sun behaviors and medical history, and provide a DNA sample. This patient-level metadata is applied to image datasets using DICOM labels. Anonymization and masking methods are applied to preserve patient privacy. A two-step annotation process is followed to label skin images for lesion detection and classification using deep learning models. Skin phenotype characteristics are extracted from images, including innate and facultative skin color, nevi distribution, and UV damage. Several algorithms will be developed relating to skin lesion detection, segmentation and classification, 3D mapping, change detection, and risk profiling. Simultaneously, explainable AI (XAI) methods will be incorporated to foster clinician and patient trust. Additionally, a publicly released dataset of anonymized annotated TBP images will be released for an international challenge to advance the development of new algorithms using this type of data.

**Conclusion:**

The anticipated results from this protocol are validated AI-based tools to provide holistic risk assessment for individual lesions, and risk stratification of patients to assist clinicians in monitoring for skin cancer.

## Introduction

The potential application of Artificial Intelligence (AI) in medicine has been increasingly explored in recent years (1). The ability of convolutional neural networks (CNN) to recognize patterns in images for medical diagnosis has surpassed the accuracy of clinical specialists in experimental settings in various medical fields (2). To date algorithms have been successfully applied to image analysis for radiology (3), cardiology (4), ophthalmology (5), and dermatology (6). However, the translation of AI into clinical practice is still in its early stages, as researchers navigate complex issues relating to patient informed consent and privacy, representative and diverse training datasets, patient and clinician trust, explainable AI, and clinical workflow (7).

In dermatology, AI has the potential to reshape diagnostic processes using numerous imaging modalities including dermoscopy and sequential digital dermoscopy imaging (SDDI), wide-field clinical imaging and total body photography (TBP), reflectance cutaneous confocal microscopy (RCM), optical coherence tomography (OCT), line-field confocal OCT (LC-OCT), and lastly digital pathology. The majority of machine learning algorithms are commonly utilized for the analysis of single lesion dermoscopic images, with notable focus on the specific task of detecting melanoma (8). A landmark study by Esteva et al., reported higher accuracy for algorithms in classifying keratinocyte carcinoma and melanoma, compared to the average accuracy score from 16 expert dermatologists (9). While several other AI models have been reported with similar high accuracy, very few have been tested in a clinical setting (10). One promising study showed support for AI-clinician collaboration, where AI supported clinician decisions scored higher accuracy than decisions made by AI or clinician alone (11).

To date, a limited number of AI models in dermatology have been applied to TBP or wide-field photography (12, 13). TBP for skin cancer monitoring provides the advantage of objectively capturing the entire skin surface and ability to compare sequential images between appointments. Furthermore, TBP requires operating software, which presents an ideal opportunity for implementation of AI support models. The appeal of applying AI tools to TBP platforms includes the potential to utilize image input from a whole patient, and to incorporate common patient-level metadata often recorded in TBP user interfaces (14). This presents an opportunity to train algorithms to consider not just a single lesion image, but also the patient’s skin characteristics and clinical background in the skin lesion evaluation process. Furthermore, algorithm output can go beyond lesion classification and extend to skin phenotype analysis, and patient risk stratification for future screening recommendations. To date there are few published articles regarding methods and standards for image annotation of TBP, incorporating skin phenotype characteristics, or on methods for preserving patient privacy when using TBP for Machine Learning.

The protocol reported within this paper was developed as part of a wider European Horizons grant (Grant ID: 965221), to design an intelligent Total Body Scanner (iToBoS) with computer aided diagnostic tools for lesion classification and patient risk profiling. This protocol describes the ‘Clinical Data Acquisition Study’, including the collection, annotation, and use of clinical data to develop the iToBoS Cognitive Assistant. This study represents a collaborative effort of a multidisciplinary team, including clinicians, software engineers, medical data annotation experts, data management specialist, bioethics experts, and patient advocates. The wider objective is to present solutions to existing challenges and aims to set standards which benefit the international AI research community. The study will include a release of an anonymized dataset for an international image analysis challenge, in which participating researchers can benchmark developed algorithms. The larger goal of this study is to foster collaborative and ethical research in the field of artificial intelligence and medical data, to facilitate advancements in technology for skin cancer detection.

## Objectives

### Primary objective

The first objective is to build a database comprising skin images enriched with annotations related to skin lesion diagnostic categories, skin phenotype information, and patient-level metadata. This dataset aims to facilitate the development of algorithms tailored for both lesion-specific and patient-level risk assessment for melanoma. This master dataset will be partitioned to create separate training, test, and validation datasets.

### Secondary objectives

The training dataset will be used for design and development of deep machine learning models with the following objectives:

1. Assess the risk associated with a captured lesion.
2. Provide diagnostic classification for the lesions presented in images.
3. Identify ‘ugly duckling’ lesions within a given individual.
4. Provide patient holistic risk assessment for future melanomas.

Concurrently, an anonymized dataset of TBP images labelled with metadata will be released for an international image analysis challenge to foster research and collaboration on the development of AI technologies for skin cancer detecting and risk profiling.

## Methods

**Figure 1:**
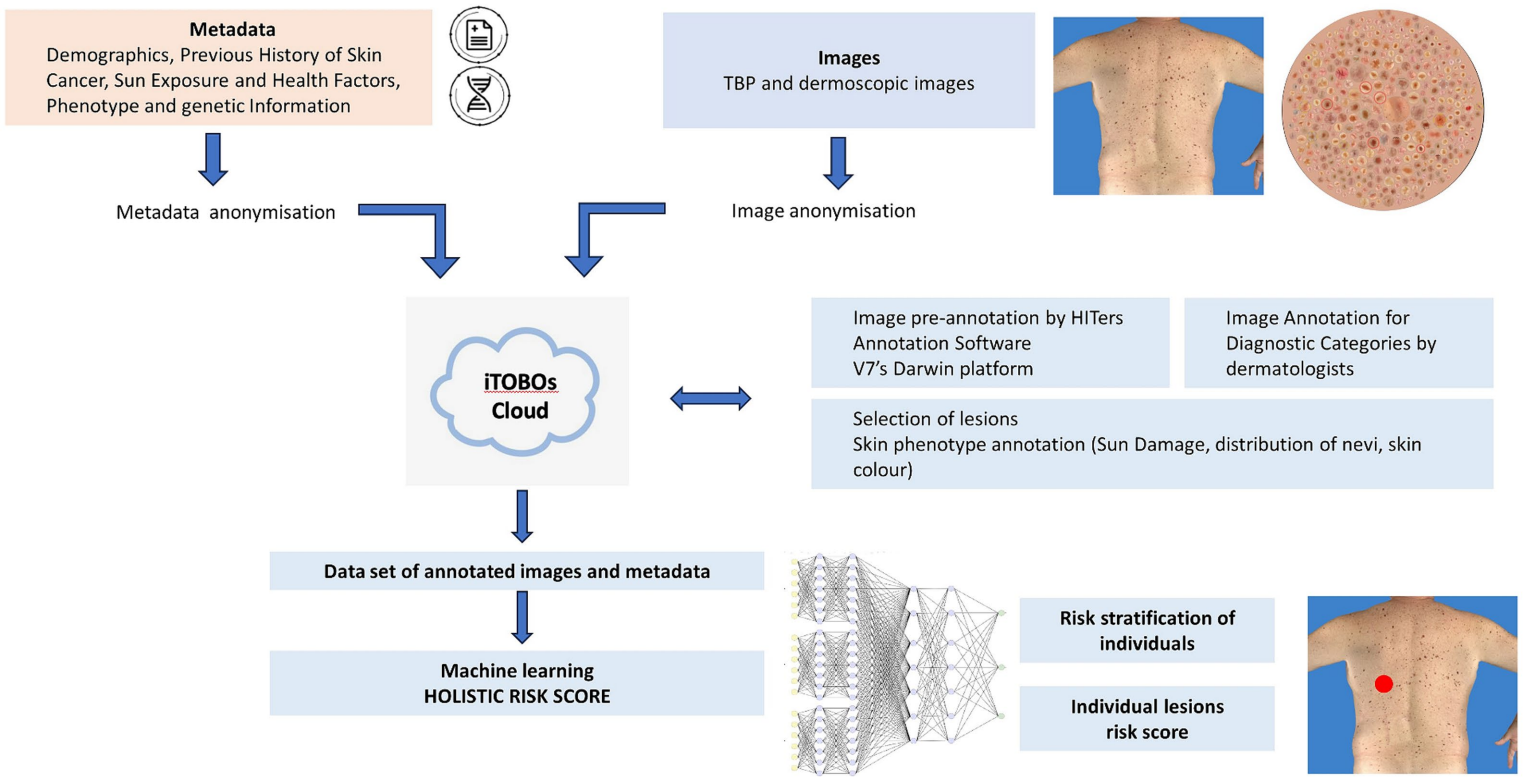
Workflow of data management for ML using multimodal data. Recruitment of patients (clinical studies); collection of images (TBP and dermoscopic images) and metadata (medical records and genetics); anonymization; annotation by HITers and dermatologists; dataset for ML training, validation and test; holistic risk score for individuals and individual lesions.

The methods described in this article pertain to procedures for data acquisition, data anonymization, image annotation, and algorithm development. A flow chart of steps involved is provided in Figure 1.

### Data acquisition

The clinical data acquisition study is carried out in two study sites: The Barcelona Hospital Clinic in Barcelona, Spain, and The University of Queensland, in Brisbane, Australia, each obtaining prospective local Human Research Ethics Committee (HREC) approval. Both studies require participants to provide written, informed consent, and collect standardized data. However, distinct protocols for recruitment and data acquisition are followed by each site.

#### Barcelona study site

Patients attending regular clinical skin examination appointments at the Barcelona Hospital Clinic are approached for participation in the study. Upon obtaining written informed consent, study participants undergo TBP imaging, complete a survey, and provide a saliva or blood sample for genetic analysis. Alternatively, they may consent to the utilization of pre-existing samples already held by the hospital.

#### Brisbane study site

Individuals who received TBP as part of a previous research study are contacted via email with a brief description of the new study. If individuals express interest, they are guided to an online consent form that outlines participation requirements. This includes consenting to the utilization of pre-existing TBP images from a prior research study, existing genetic samples, and the prospective completion of an online survey. If individuals consented to participate, a link to the online survey is provided via email for completion.

#### Participant timeline

Participants for the Australian site were enrolled between October 2022 and April 2023. Participants granted consent for the utilization for TBP images captured with the VECTRA WB360 during previous research visits spanning from September 2016 to February 2020.

Enrolment of participants at the Spanish site commenced in January 2023 and is currently ongoing, with an expected finish date in mid-2024.

#### Sample size

Optimal sample sizes for AI-based image classification tasks are difficult to estimate. Therefore, for the purpose of this study, a target sample size of 500 patients will be recruited. It is expected that 15% (*n* = 75) will be low/average risk for melanoma, 50% (*n* = 250) will be moderate/high risk, and 35% (*n* = 175) will be very high risk. The majority of the patients will have up to six sequential TBP captures, taken at intervals of every 6 to 12 months. From each TBP capture, an average of 500 image tiles (8 cm × 10 cm) will be produced, annotated and available for development of the photodamage CNN algorithm.

Over a 2 years period, considering the melanoma incidence in Queensland (Australia) (15), we anticipate incidence rates of 0.3, 1.2, and 2.8% for individuals classified under low/average risk, moderate/high risk, and very high risk, respectively. Therefore, we expect 8 annotated melanomas (*n* = 0.2, *n* = 3, *n* = 5 in sequence). Correspondingly, it is expected that there will be 67 keratinocyte cancers for every melanoma diagnosed (16). Therefore, we expect 549 keratinocyte cancers (13 in the low/average risk group, 201 in the moderate/high risk group and 335 in the very-high risk group).

With respect to benign lesions, the median general population total body nevus counts >2 mm in diameter in the Queensland general population are 32 nevi per person (17). Higher counts are expected for those at higher risk of melanoma with a median number of nevi >2 mm in diameter reported in a Swiss cohort at 189 per person (18). We therefore conservatively estimate those at moderate/high risk to have a median around 80 nevi >2 mm in diameter. Hence, a total of approximately 66,475 nevi (2,400, 20,000, 33,075 respectively) will be annotated across 500 individuals at one time point. We estimate a ratio of 1:4 for nevi:other benign lesions (19), and therefore estimate an additional 265,900 other benign lesions for annotation.

#### Image acquisition

All participants underwent 3D total body photography using the VECTRA WB360 whole body 3D imaging system (Canfield Scientific Inc., Parsippany, New Jersey, United States). In Barcelona, TBP was conducted prospectively (after consent signed); in Brisbane retrospective TBP image archives were accessed after consent to participate was provided. The VECTRA WB360 system is a framework of 92 cameras, in which a study participant is asked to stand, holding a specific anatomical pose. All 92 cameras simultaneously capture images of the patient, and software is used to construct a 3D avatar from those images. An attached dermoscopy camera (Visiomed D200e dermatoscope, Canfield Scientific), allows additional high-resolution images of single lesions to be recorded and mapped to the 3D avatar. This 3D Imaging System enables the objective documentation of all skin lesions (excluding scalp, soles of feet and any skin covered by underwear) and facilitates the monitoring of changes over time.

#### Metadata acquisition

A 45-item survey was designed to collect information relevant to melanoma risk, while maintaining a low risk of being individually identifiable. The questions included were based on previously validated surveys to include known risk factors for melanoma (20–22). The questionnaire collected four categories of information:

1. Demographics: age, year of birth, sex, country of birth, current country of residence, previous country of residence, height (cm), weight (kg), marital status, education level, occupational status, and ancestry.
2. Previous History of Skin Cancer: number of previous melanomas/BCCs/SCCs and age of first melanoma/BCC/SCC, number of benign melanocytic lesions excised, family history of melanoma.
3. Sun Exposure and Health Factors: occupation (indoor/outdoor), frequency of clinical skin checks, history of sunburn, sunbed use, sunscreen use, smoking status and history, ongoing (significant) medical treatment, e.g., immunosuppressants.
4. Phenotype Information: hair/eye/skin color, skin type (e.g., easily burns/tans), freckle and nevi density.

Survey answers are collected directly from study participants, and stored using the online web-based application, REDCap (Research Electronic Data Capture). REDCap is highly secure and compliant with standards set by the Clinical Data Interchange Standards Consortium (CDISC). All data collected in this study will be entered directly into an eCRF (electronic case report form) in REDCap under a designated Study ID code for each participant. A separate confidential record of personal details (name and contact details) will be kept on an enrolment log, separate from the eCRF.

### Data anonymization

#### Image anonymization

To effectively anonymize the 3D TBP images for annotation, the original 2D macro images are divided into small tiles of approximately 8 cm × 10 cm, padded with a 5% overlap in each direction, to ensure coverage of all lesions. For each tile, a set of tile masks are also produced representing (a) the unique regions of the tile for which the tile has the best (most orthogonal) view of those regions, (b) the area of the tile excluding any padding, and (c) the initial AI-based lesion detection produced by the VECTRA WB360 (13). Average resolution and primary anatomic location are also recorded for each tile. Anatomic location is specified as one of ten categories: Torso (front & back), Left & Right Arm (Upper & Lower), Left & Right Leg (Upper & Lower).

For further anonymization, the patient’s face and head are automatically identified and masked out in any tile. Tools are also available to paint any additional areas that may be identifiable, such as tattoos.

The tile masks, lesion auto-detection, and associated metadata are saved for direct uploading to the annotation software, *Darwin* platform (V7 Labs, London, United Kingdom). Sufficient metadata on each tile are recorded so that any later annotation on the tiles can be mapped back to the 3D TBP avatar image.

#### Metadata masking

Before uploading any patient data files to the iToBoS Project cloud, different masking and/or anonymization methods are applied to reduce the risk of patient re-identification. The metadata collected via participant surveys are exported from REDCap into both CSV files and in DICOM metadata headers. The IBM masking tool (23) is used to mask this metadata for both file types in a consistent manner across all files belonging to the same patient.

The main types of fields that are currently masked using the IBM masking tool are:

1. Patient related IDs—Hospitals store information that connects these IDs to a specific patient. They are masked using an irreversible format-preserving tokenization.
2. Dates - Since visit dates can also potentially re-identify specific individuals, noise is added to all dates within a specified range (of 60 days). In order to preserve the order (and gaps) of the dates to enable correct analysis, the noise is computed per patient and applied consistently to all dates belonging to a specific patient.
3. Ages—Ages above 89 are rounded down to 89 as per HIPAA requirements.

#### Artificial intelligence privacy

As part of the project’s dedication to patient privacy and compliance to regulations, once the AI models are trained and validated we also plan to apply two additional tools for anonymizing the AI models themselves (24) and for minimizing the data collected for analysis in accordance with GDPR’s data minimization requirement (25).

### Image pre-annotation

#### Pre-annotation workforce

The *Isahit* workforce is composed of HITers (stands for Human Intelligent Tasks workers) responsible for annotating skin lesions and sun damage levels on each image tile. This workforce is composed of women from Africa, Southeast Asia and South America, who receive extensive training and support in image annotation and digital skills.

Researchers from the Barcelona and Brisbane clinical sites provide a set of guidelines including text, images, and videos to train the HITers for the pre-annotation process. Initially, HITers annotate multiple test datasets to facilitate the training process. Clinicians can then review and provide feedback after each dataset, and this iterative process continues until the annotation achieves an acceptable quality and accuracy of >80%. A lower threshold of accuracy for pre-annotation is permitted as opposed to annotation for lesion diagnostic categories, as the potential impact on ML is less consequential (e.g., estimate of sun damage vs. detection of skin cancer).

After training is complete, each image annotated by the HITers is reviewed by a second person from the Isahit team to ensure protocols are followed correctly and accuracy is maintained. Initially, all images are additionally reviewed by a medical researcher from the clinical sites until quality and accuracy is acceptable. After this, 10% of images will continue to be reviewed by the clinical sites to ensure ongoing accuracy of >80%.

#### Annotation software

V7’s *Darwin* platform facilitates the viewing and complex annotation of the skin image tiles. The platform enables users to designate a workflow in which images are assigned to various HITers and subsequently reviewed by clinical sites. Users across this workflow can view and edit image labels simultaneously, while allowing reviewers to provide comment, explanation and reject incorrectly annotated images.

**Figure 2:**
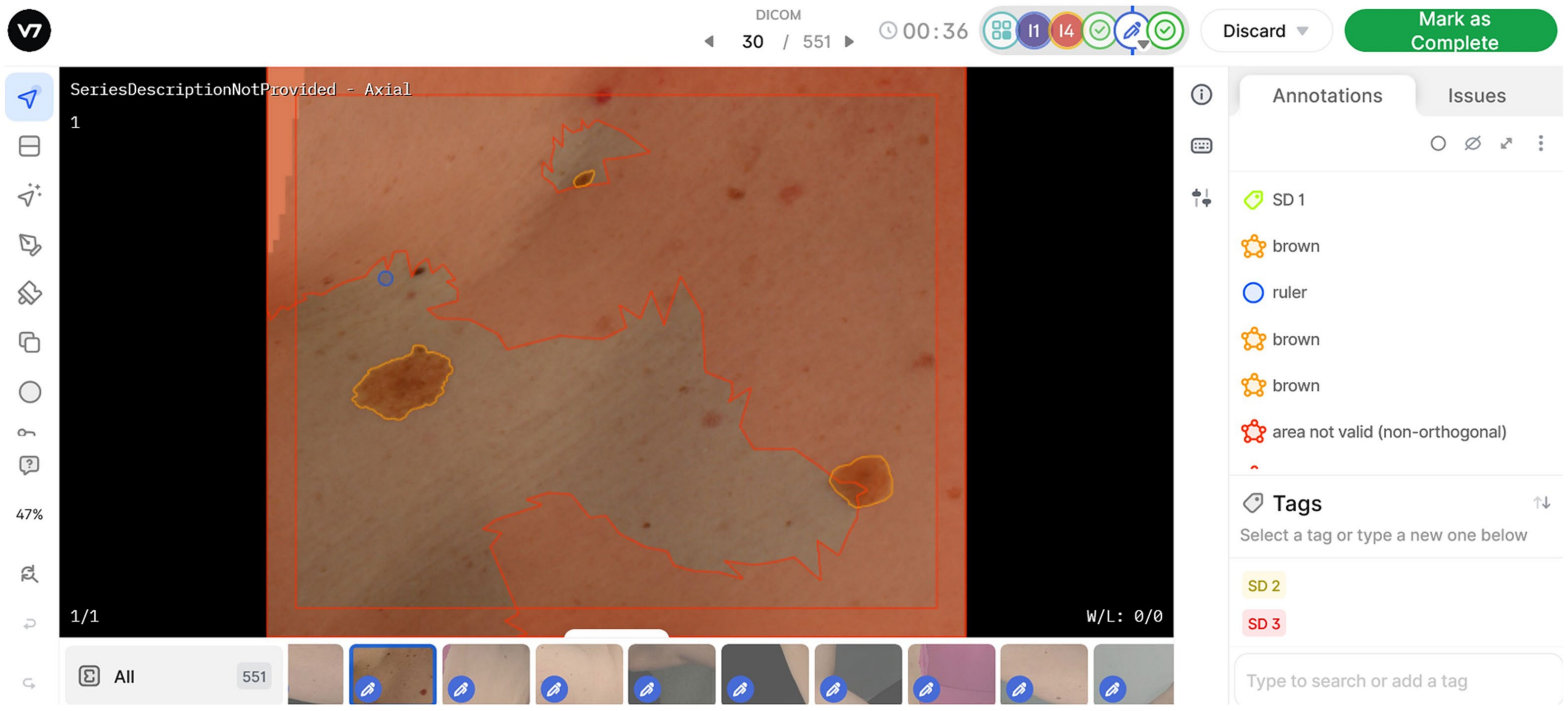
Work view in V7 software, enabling annotation and classification of lesions and sun damage. Symbols on the left side panel represent annotation tools available, allowing users to generate new annotations or edit existing lesion masks. All annotations or masks applied to the image are visible on the right-side pane. The red areas on image tiles are denoted as “area not valid,” as they are better represented for annotation in another image tile. One sun damage (SD) tag can be applied per image based on assessment of the valid area.

Each dataset of image tiles represents one VECTRA TBP capture for one patient. Once uploaded to the V7 Darwin platform, any detected lesions are auto-annotated with an ‘*unlabelled*’ tag. Every image tile contains a circular digital ruler with a standardized diameter of 2.5 mm, enabling HITers to measure lesions during the annotation process. The HITers are instructed to review the auto-annotated lesions for falsely pre-tagged lesions, either because they measure <2.5 mm, or because they are artefacts (e.g., umbilicus, hair, scratch/scar etc.). Likewise, annotators will need to adjust segmentation borders when multiple lesions have been grouped together by the auto-annotating software. HITers are then instructed to update the ‘unlabelled’ tag to a lesion color category (Brown, Pink/Red/Purple, Black, Blue, White, Skin Color, or Combination). Lastly, HITers will categorize the average sun damage for each image tile as (1) None/Mild, (2) Moderate, or (3) Severe. Annotators are provided with image examples to use as reference in their assessment. An example of the Darwin user interface is displayed in Figure 2.

### Image annotation for diagnostic categories

A second round of annotation is completed by expert dermatologists, or dermatologically trained clinicians, for the diagnostic categories displayed in Table 1. This process is to be completed by the two recruitment sites (Barcelona and Brisbane), as well as an additional clinical site, the University of Trieste, Trieste, Italy.

**Table 1:** Final diagnostic categories for lesion annotation, with shorthand acronym in brackets.

Diagnostic categories for annotation	Shorthand
Benign melanocytic lesion	BM
Suspicious melanocytic lesion	SM
Melanoma	MM
Solar Lentigo	SL
Seborrheic Keratosis	SK
Angioma	AG
Actinic Keratosis/Intraepidermal carcinoma	AK/IEC
Dermatofibroma	DF
Basal Cell Carcinoma	BCC
Squamous Cell Carcinoma	SCC
Inflammatory Lesion	IL
Collision Tumor	CT
Unknown	UN
Other	OT

This is conducted using V7’s Darwin platform and verified through VECTRA WB360 DermaGraphix Software. The image tiles annotated in the Darwin platform are reconstructed and projected back to the 3D TBP image for native visualization in the DermaGraphix software. This software allows the expert dermatologist to view the annotated lesions with the 3D avatar as a reference, with the ability to further annotate each lesion into diagnostic categories. The diagnostic classes for annotation were selected to be appropriate for the lower resolution of TBP images (as compared to dermoscopy). The diagnostic hierarchy available for melanocytic lesions (benign, suspicious, melanoma), reflect increasing levels of confidence in lesion categorization, including the pre-annotation of color.

### Skin phenotype annotation

#### Sun damage

As described in the previous section, the HITers workforce used a photo-numeric scale to evaluate the level of sun damage for each image tile during the pre-annotation step. This scale used three categories of sun damage: none/mild, moderate, and severe. Each rater was provided with written instructions, including several image examples, on how to rate photodamage. Each image tile is assigned only one sun damage label, with the highest sun damage level being designated if multiple levels are evident within the tile. Sun damage is then summarized for each individual patient by calculating the percentage of skin surface categorized for each level of sun damage, as well as providing sun damage scores for anatomical sites, i.e., arms, legs, and torso.

#### Nevi distribution characteristics

Implemented within the Canfield VECTRA DermaGraphix software, a Convolutional Neural Network (CNN) offers the probability assessment for each lesion, indicating the likelihood of it being a nevus. This algorithm was trained using ratings from an expert dermatologist as the gold standard (13). In addition, the longest diameter and 3D spatial coordinates of each lesion are calculated. For this study, all lesions with a diameter ≥ 3 mm with a nevus confidence >0.8 were considered nevi. This enables overall nevus count for the patient, as well as nevus distribution across anatomic sites.

#### Skin color measurement

Average skin color can be automatically measured across the 3D TBP image as the individual topography angle (ITA), calculated in the L*a*b* color space. A recent study in a highly sun-exposed population showed that most people had no photodamage on their lower torso and lower back third (26). Therefore, to calculate innate skin color the average of the non-lesion skin color from these locations is calculated. To estimate facultative skin color the average non-lesion skin color is calculated for the dorsal side of lower arms. The L* and b* values may be adjusted using a regression equation if it results in higher agreement with gold standard colorimeter values.

### Algorithm development

Algorithms will be developed to apply automatic annotations on previously unseen images acquired with the VECTRA WB360 and identify evolving changes in a patient’s skin images over time. Task specific algorithms include lesion detection and segmentation on the image tiles, inter-exploration lesion-matching, ugly duckling detection, classification of the detected lesions, intra-exploration lesion matching and lesion change detection. From the results of these algorithms, imaging phenotypes to compute the lesion risk assessment are extracted.

#### Data splitting

The annotated image tiles are divided into three distinct datasets (training, validation, and test datasets), for AI-based model development and evaluation process. This allocation ensures that the models are trained on a substantial portion of the data (training set), evaluated and tuned based on the validation subset, and ultimately tested on unseen data (test set) to estimate its real-world performance accurately.

The methodology employs a stratified randomization technique with 65% of the data allocated to the training set, 20% to the validation set, and the remaining 15% to the test set. Stratification is used to ensure all diagnostic classes (Table 1) are sufficiently represented in each dataset. This is vital to prevent potential class imbalances that could adversely affect the models’ performance and generalization.

#### Lesion detection and segmentation

For this task, a three-step approach is used to tackle lesion detection and segmentation. Given that image tiles may include background other than skin, images are pre-processed to obtain a corresponding skin mask. This is achieved by applying image thresholding within the HSV (hue, saturation, value) color space, followed by the application of morphological operations to enhance and clean the segmentation result. The resulting skin mask is applied to the final lesion segmentation, to eliminate the inaccurate detections (outside the skin region).

For lesion detection, the YOLO (You Only Look Once) model is employed (27), customized for our specific dataset. To expedite the model’s convergence and improve its performance, the training process is initialized using pre-trained weights on the COCO (Common Objects in COntext) dataset (28). This approach leverages the knowledge and features learned from a broader dataset and fine-tunes the model for our dataset. By initializing with pre-trained weights, the model is built on a solid foundation and already possesses an understanding of basic object features, allowing it to adapt more efficiently to our data. This transfer learning strategy not only accelerates the training process but also often leads to better performance compared to training from scratch, particularly when working with limited data.

**Figure 3:**
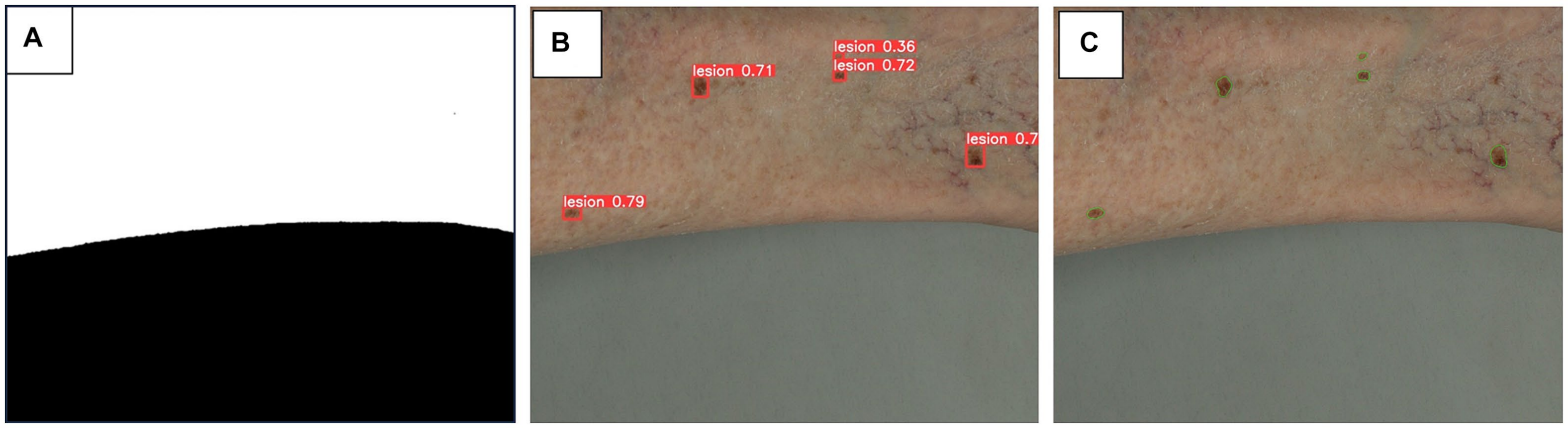
Algorithm steps for skin masking **(A)**, lesion detection **(B)** and lesion segmentation **(C)**. A three-step approach is used for lesion detection and segmentation. **(A)** as image tiles may include background of non-skin area, a skin mask obtained by thresholding of HSV color space, is applied to minimize inaccurate detections on non-skin area. **(B)** YOLO (You Only Look Once) (27) model, pretrained on COCO (Common Objects in Contexts) (28) dataset, is adapted to our datasets to detect skin lesions, depicted as bounding boxes. **(C)** The bounding box predictions are used as prompts for segmentation, leveraging SAM (Segment Anything Model) (29) model to provide precise segmentation of skin lesions.

The bounding box predictions provided by the lesion detection model are used as initial prompts for subsequent instance segmentation, for which we leverage the Segment Anything Model (SAM) (29) performance. SAM is a state-of-the-art instance segmentation model known for its accuracy and robustness. Its impressive zero-shot performance, often comparable to or better than fully supervised models, highlights its versatility and potential for various applications. An example of the results of this three-step approach is presented in Figure 3.

#### Inter-exploration lesion matching

The objective of this task is to pinpoint the most optimal view, specifically the one that appears most orthogonal to the camera, for each detected lesion on the patient. With the help of the patient’s body geometry (i.e., acquired and properly anonymized 3D model), along with the system’s geometric calibration parameters, informed predictions can be made regarding the location of each lesion within the image tiles and approximate their image coordinates in each tile (with a margin for calibration error). To refine the predicted location based on the system geometry, we take advantage of the image information and perform feature matching in a subsequent step, either based on lesion or image features, to obtain a more precise lesion location.

**Figure 4:**
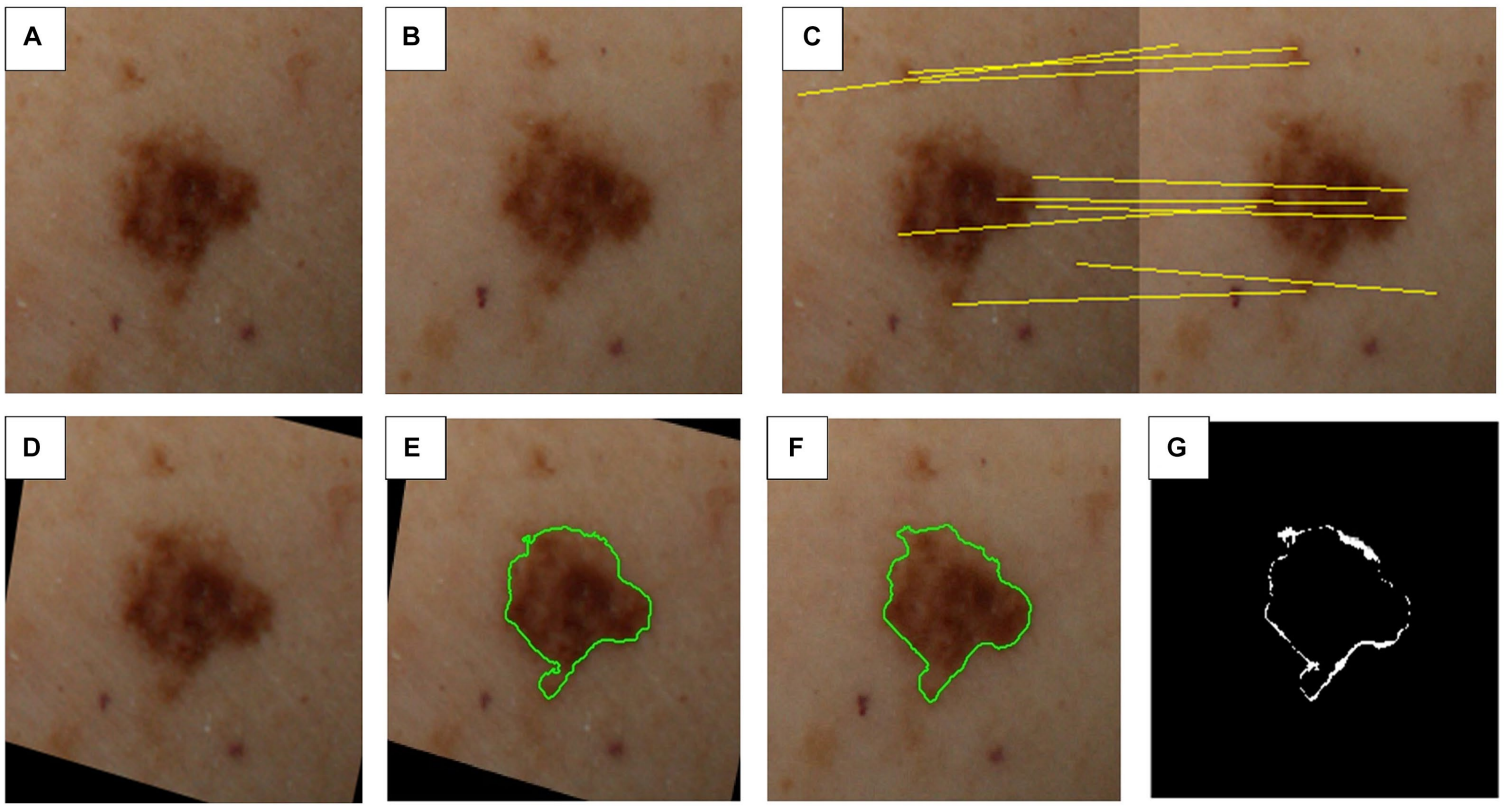
Steps involved in inter-exploration lesion matching and change detection. **(A)** First image exploration (i.e., image capture). **(B)** Second image exploration (e.g., 6–12 months later). **(C)** Feature matching between sequential explorations. **(D)** Transformed first image exploration to match second image exploration. **(E)** Segmentation mask for first image exploration. **(F)** Segmentation mask for second image exploration. **(G)** Detected differences between explorations.

Once all the views corresponding to a particular lesion are identified, the best view is selected based on its positioning within the patient’s 3D model in relation to the camera system’s geometry. This optimal view for each lesion is then extracted from its respective image tile, resulting in a collection of cropped individual lesion images. This process is illustrated in Figures 4A–D.

#### Super-resolution

Machine learning models, particularly deep learning models, trained on high-resolution data tend to perform worse when applied to real-world scenarios, where image quality may vary. Methods to enhance fine details and subtle features in lower-resolution images allow for more precise analysis. Super-resolution techniques can significantly improve the visual quality and sharpness of images. By applying this technique to the collection of cropped individual lesion images, high-resolution versions of the images can be generated, which are visually more appealing and detailed, and can improve the performance of the trained models.

For image super-resolution, the GAN prior embedded network (GPEN) (30) is used on the lesion crops. We fine-tune the GPEN model, which initially utilizes pretrained weights from the FFHQ (Flickr-Faces-High-Quality) dataset (31) on our own generated data, including artificially degraded dermoscopy images from the International Skin Imaging Collaboration (ISIC) challenge dataset (32). This degradation process includes resizing, blurring, and adding noise to the original images. It enables the model to be fine-tuned to match our specific super-resolution needs and achieve image quality as close as possible to dermoscopy.

#### Skin lesion classification

The outcomes of the previous steps generate datasets containing cropped single-lesion images. To make use of this data and their corresponding diagnostic annotations, we employ a multi-class classifier to automatically identify skin lesions. This classifier categorizes the skin lesions into the classes provided in Table 1. Given that the provided images are approximately 210 × 210 pixels in resolution, we opt for smaller deep learning models designed for handling such dimensions efficiently.

Given the established success of EfficientNet in tasks related to skin cancer detection, our approach involves the utilization of EfficientNet models, specifically choosing from smaller variant models B0, B1, B2, or B3 with fewer parameters for the analysis of our dataset. EfficientNet have demonstrated notable efficacy in achieving accurate results while managing computational efficiency. The flexibility of selecting among these variants allows us to tailor the model to the specific characteristics of our data, ensuring optimal performance in skin lesion diagnosis.

The choice of the final EfficientNet model is still pending, awaiting experimental evaluation to determine the most effective model considering the image resolution. However, the smaller EfficientNet models, in comparison to cutting-edge alternatives, are considered well-suited for application in TBP. Their smaller size not only aligns with the need for speed in this task but also ensures efficiency without compromising performance in the diagnosis of skin lesions.

#### Comparative analyses and ugly duckling detection (nevus phenotype)

Most individuals exhibit a predominant type of nevus (e.g., signature nevus), sharing a similar clinical (or dermoscopic) appearance. Lesions outside of this common pattern in a given individual (i.e., the ugly duckling), must be considered with suspicion, even if it does not fulfill the ABCDE or dermoscopic melanoma-specific criteria. Conversely, an atypical lesion may be completely normal in an individual whose skin is covered with similar lesions in the context of atypical mole syndrome (33, 34). The lesion classification model’s feature extraction capabilities are harnessed to identify “ugly ducklings.” This is achieved by computing the features of individual moles and comparing them with those of other lesions within the same exploration.

Upon mole detection, feature extraction is applied to all potential lesions. This entails computing the output of the last layers of the classification CNN model. To streamline the problem and retain only the most informative features for lesion description, Principal Component Analysis (PCA) (35) is carried out. Subsequently, the L2 norm is employed to calculate a similarity score between each pair of lesions. Any lesion that significantly differs from the rest (indicated by a low similarity score relative to other lesions) is flagged as a potential “ugly duckling.”

#### Lesion change detection

A similar strategy used in inter-exploration lesion matching is applied to match the same lesion across different image captures of the same patient. First, a 3D-based hint for matching skin lesions is computed, and then further refined through a subsequent image-based matching phase involving candidate skin lesions located within the predicted area.

The VECTRA WB360 3D model is transformed into a generic human avatar by fitting a Parametric Body Model (PBM) to the patient’s scan. The PBM serves as an intermediary layer between different explorations (i.e., image captures), allowing lesion positions to be mapped to the avatar and then matched to the next exploration. This position estimation is further refined by performing feature matching on the corresponding images.

**Figure 5:**
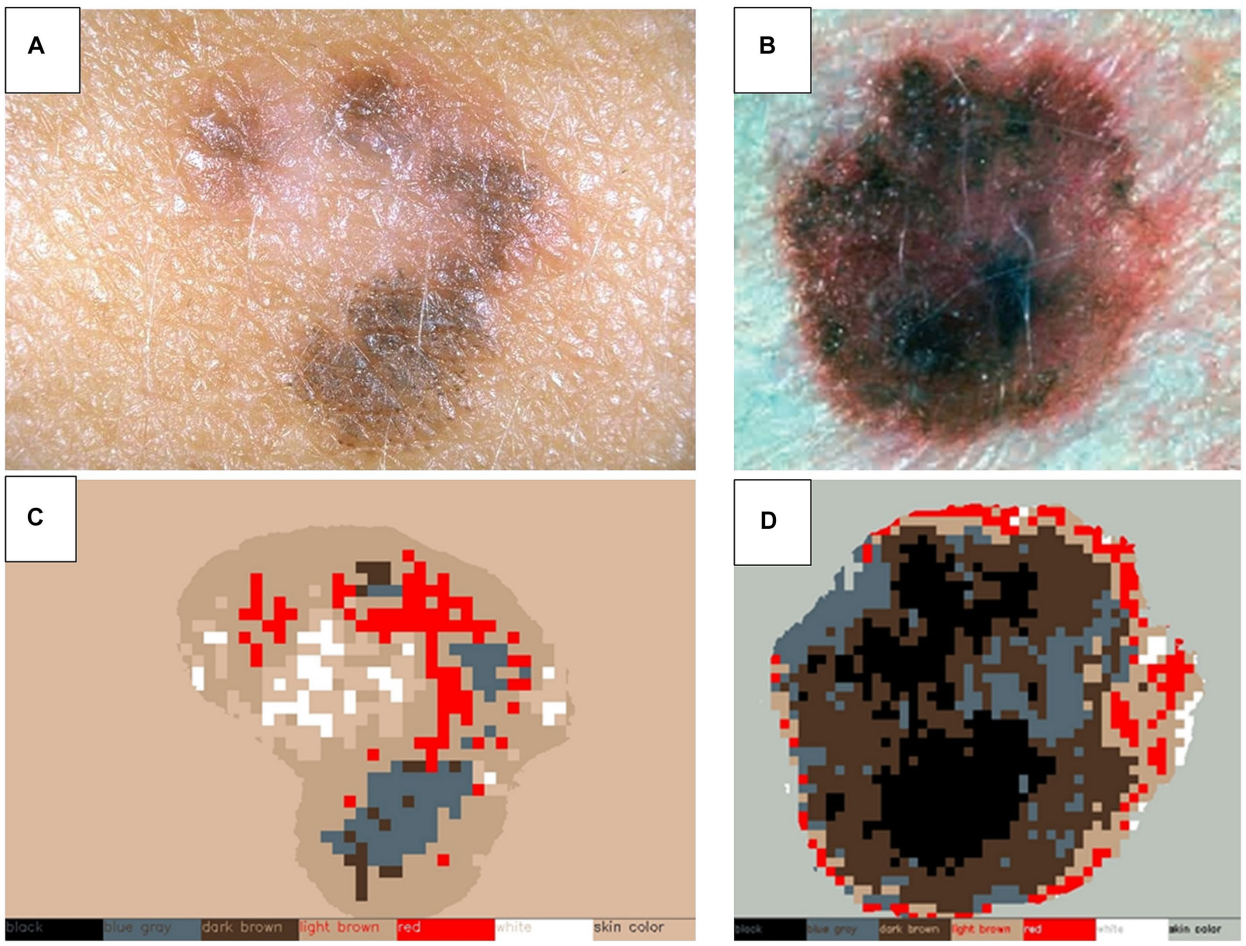
Color detection of selected skin lesions. Two examples of original skin lesion images **(A,B)**, and subsequent color detection applied **(C,D)**. This step is used to compare sequential explorations of lesions to detect changes in color.

Upon identifying lesion matches between the two explorations, changes in color, shape, and size are assessed, as well as pinpointing any newly appearing lesions. To analyze shape and size variations, the segmentation masks computed in the previous algorithms are registered and compared, as depicted in Figure 4. For color changes, the RGB intensities detected within the lesion mask are categorized into color groups including black, white, blue-grey, light brown, dark brown, and red, as illustrated in Figure 5. This categorical segmentation is then compared between both time points, yielding a quantification of color changes.

#### Genetic risk assessment

Patient samples (either blood or saliva) are used to extract DNA for genetic risk assessment for melanoma. Previous Genome Wide Association Studies (GWAS) have identified several single nucleotide polymorphisms (SNPs) associated with melanoma risk (36, 37). Most of these SNPs are located in genes associated with skin pigmentation, nevus lifecycle, DNA repair and telomere length. Each individual SNP represents only a small incremental change in melanoma risk, however the cumulative effect, calculated as a polygenic risk score (PRS), is estimated to contribute as high as 30% of melanoma susceptibility (38). Previous methods are used to calculate the PRS based on the reported incremental contribution of SNPs as genetic risk factors (36, 39). Additionally, the applicability of AI-based methods for genetic risk assessment will be explored. Preliminary results show that AI-based methods are quite effective in discovering potential complex interaction patterns among a large number of SNPs.

In addition to SNP analysis for polygenic risk, participants who meet familial melanoma testing criteria (40) are offered genetic testing for genes associated with high risk of hereditary melanoma. Pathogenic variations in these genes are associated with a >50% lifetime risk of melanoma (41). Inheritance of these variations follow an autosomal dominant pattern, meaning first-degree relatives of a carrier will have a 50% chance of harboring the same pathogenic variation. The detection of a pathogenic variation will be included as patient metadata to contribute to risk profiling. The actual variant details will not be released by the clinical sites, only the presence of a high-risk variation will be reported (e.g., yes/no).

#### Patient risk assessment

A cognitive assistant is built using AI-based methods for providing representative estimates of melanoma risk to the clinicians, combining different data modalities. The incremental contribution of individual risk factors is combined, including clinical metadata (collected in participant surveys), phenotype characteristics (extracted from total body photography), and genetic risk (calculated PRS and presence of high-risk pathogenic variations). Previous reported weightings for lifestyle and clinical risk factors for melanoma, collected in participant metadata surveys are applied (42, 43). Risk models are then fine-tuned using state-of-the-art data-driven AI-based analysis methods, as non-negative neural networks, to reveal new possible factors that may contribute to risk through the interaction of multiple attributes.

The final individual melanoma risk scores, derived from various data modalities across multiple patient visits, will be combined to generate a global melanoma risk estimation. This holistic assessment offers clinicians the capability to review and thoroughly evaluate the factors contributing to the reported risk score, facilitating a comprehensive understanding of the patient’s melanoma risk.

#### Lesion risk assessment

To assess the risk associated with a lesion, the outputs from previously described models are essential. These algorithms provide information of lesion size, color, diagnosis and skin tone used in the risk assessment algorithm as well as patient risk scores. This lesion risk assessment algorithm furnishes a final risk score ranging from 0 to 1 for each identified lesion. This score is normalized based on the severity of the skin disease characteristics detected, for example between seborrheic keratoses and melanoma. A score of 0 indicates healthy skin, while a score of 1 signifies a high risk of skin cancer. The algorithm will incorporate knowledge of specific skin diseases and conditions to evaluate risk, leveraging well-established characteristics in this assessment.

#### Integration of explainable AI

Explainable AI (XAI) techniques are employed to gain insights into the decision-making processes and rationales of underlying AI algorithms used for diagnostic predictions. While local explainability methods, like layer-wise relevance propagation (44), are utilized to identify the most significant input features (i.e., pixel values) for the diagnostic AI algorithm’s predictions, global XAI approaches, such as spectral relevance analysis (45), reveal global prediction strategies employed by the model.

Furthermore, concept based explainability methods, e.g., concept relevance propagation (46), combine both perspectives, and explain individual prediction with human-understandable concepts employed by the diagnostic AI tool. This further allows the automated generation of XAI-based metadata, e.g., based on the existence of certain concepts, which, for instance, might align with domain expert knowledge. Alternatively, the occurrence of artefact-related concepts (e.g., skin markings), can be used for the automated flagging of data samples.

Moreover, as suggested in the “Reveal to Revise”-framework (47), explainability techniques are seamlessly integrated into the AI model development life cycle. This involves leveraging metadata generated through XAI to iteratively identify and address model irregularities. For instance, the approach involves rectifying issues such as the utilization of data artefacts. This integration enhances the robustness and trustworthiness of the employed AI models over time.

### International skin image analysis challenge

In recent years, it has been a common research practice to organize international competitions or challenges in which the algorithms of different researchers can be benchmarked on publicly released datasets.

Over the period of the iToBoS project, the wider consortium will organize two competitive challenges where world-leading groups can participate in solving new problems on: (1) lesion detection and boundary segmentation in regional (total) body images, and (2) on lesion classification.

These challenges will facilitate advancements in the development of AI and computer aided diagnostic tools and contribute to the knowledge dissemination in the field. A selection of non-identifiable skin images prepared in this study may be included in larger datasets for the analysis challenges. In this instance, minimal clinical non-identifiable information will also be included (age, sex, melanoma history, hair and eye color and body site of image), and whether certain genetic changes, i.e., risk associated genotypes are present.

### Data management and quality assurance

Data management in iToBoS is controlled by a pre-defined Data Management Plan, which describes how we collect, store, organize, maintain, retrieve, and use information in a secure and effective manner. The primary platform of data management is the iToBoS cloud—a private cloud hosted within the Hungarian HUN-REN research cloud, which provides storage, compute and GPU capabilities for the various processing components of the project. All data is ultimately stored and processed in or via the iToBoS cloud. The Storage Cloud is based on Nextcloud software. Access to Nextcloud is managed by internal authentication and authorization backend. New users can be added by the administrators. The operating system of the virtual machines and the data stored in the cloud are encrypted leveraging the LUKS (Linux Unified Key Setup) technology provided by the Compute Cloud service. The Compute Cloud runs on OpenStack. The cloud infrastructure is accessible only by the project members using SAML (Security Assertion Markup Language) authentication. The compute cloud can host the necessary virtual machines for the project. The virtual machines can be run in a private network, which is not accessible from the outside world. It is also possible to create multiple private networks to separate the virtual machines within the project. The cloud compute provides a firewall which by default blocks all incoming traffic to the virtual machines. The virtual machines can be accessed using SSH (Secure Shell) protocol, which is protected by public key. The iToBoS cloud also provides GPUs for AI inferencing tasks.

### Data monitoring

#### Patient consent, metadata, and image acquisition

Site reviewers will randomly select 10 participant visits on a monthly basis to retrospectively check for adherence to protocols in regard to:

- Patient consent for completeness and accuracy.
- Input of data into eCRFs for completeness and accuracy.
- Images produced by VECTRA in regard to positioning of participants, consistency, and image quality.

Feedback from monitoring visits is to be provided to clinical sites quarterly.

#### Image annotation

The first subset of participant image datasets pre-annotated by the ISAHIT workforce are 100% reviewed by the clinical sites for accuracy, with feedback provided after each dataset is reviewed. This process is repeated until accuracy has reached at least 80% agreement between ISAHIT annotators, and the clinical sites. After which, 10% of datasets pre-annotated by ISAHIT will receive clinician review, with direct feedback provided if annotation is rejected.

For lesion classification annotation performed by expert dermatologist, an initial review stage will take place after each clinical site has annotated 10 participant datasets. Each dataset will be reviewed by a different clinical site to calculate agreement between two expert dermatologists. If agreement is over 95%, then lesion classification annotation will continue without further review. If agreement is under, then the panel of dermatologist annotators will meet to discuss cases in disagreement. Dermatologists will then annotate and provide review for another 10 patient datasets and repeat this process until agreement is over 95%.

### Ethics and dissemination

Clinical data acquisition studies received Human Research Ethics Committee (HREC) approvals from The University of Queensland HREC (2022/HE001866) for the Brisbane, Australia site, and from the Hospital Clinic of Barcelona HREC (HCB/2022/1051) for the Barcelona, Spain site. The study has been registered with ClinicalTrials.gov (ref NCT05955443). The study was conducted in accordance with the principles of Good Clinical Practice. The study was designed following the ethical principles of the Declaration of Helsinki from 1964. Results will be published in peer-reviewed journals and disseminated at international scientific meetings.

### Patient and public involvement

The iToBoS Consortium include partnership with the Melanoma Patient Network Europe (MPNE), to ensure direct consultation and dissemination of research to melanoma patients and the public. MPNE reviewed this protocol prior to implementation to provide feedback on the study design on behalf of the melanoma patient network. Furthermore, MPNE are running a series of workshops for MPNE members over the course of the project, which include presentations and questions time with iToBoS Consortium researchers.

Additionally, iToBoS Consortium partner The University of Queensland hold regular consumer forums, to provide a platform for researchers, advocacy groups and consumer representatives to meet and exchange ideas. These forums also include presentations on recent research developments related to melanoma, with ample time allocated for consumer questions and discussion (48).

## Anticipated results

At the completion of this study, we anticipate having a comprehensive annotated dataset of total body photography images, anonymized, and labelled with patient-level metadata. Images will be annotated with information relating to skin phenotype and lesion diagnostic categories. Patient-level metadata will enable machine learning protocols to evaluate individual risk for melanoma. The AI tools we anticipate will include algorithms for lesion detection, segmentation, matching (from previous imaging), change detection, ugly duckling assessment, and lesion diagnostic category. All these algorithms contribute to the final output of lesion risk assessment. A second holistic patient risk algorithm will be developed using patient-level metadata and skin phenotype information.

Furthermore, with research partners dedicated to the development of explainable AI (XAI) procedures, we anticipate incorporating XAI description with all risk assessment algorithm output. Lastly, it is our objective to contribute annotated datasets to two international computer skin image analysis challenges to advance further developments in this field and promote wider collaboration.

## Discussion

To our knowledge, this protocol is the first to describe the multiclass annotation of total body photography (TBP) for both skin lesion and phenotype characteristics. This includes DICOM labelled patient-level metadata designed for machine learning application. Algorithms developed with these datasets will address several clinically relevant gaps that previous skin lesion classifier algorithms often lack (49). The majority of algorithms to date are trained to consider a dermoscopic image of a lesion, in isolation of any clinical background. This has negatively impacted the translation of this technology into clinical practice. In a real-world scenario, a dermatologist would consider a patient’s medical history, phenotype (including skin/hair/eye color, nevi characteristics, and UV damage), review sequential imaging to detect lesion changes, and apply ‘ugly duckling’ methods, which draws attention to lesions that are unlike others on a given patient. The aim of this protocol is to develop computer aided diagnostic (CAD) tools capable of providing holistic risk assessment of both patient and individual lesions.

One advantage this protocol presents is the prospective collection of TBP from patients at various risk of melanoma, providing a representative image dataset of the entire skin surface for both general and higher risk populations. To date, algorithms for skin lesion classification are often developed using retrospective public datasets of mostly dermoscopy images, which may be restricted to ‘interesting cases’ and lack generalizability to day-to-day clinical practice (6). Furthermore, an algorithm applied to TBP or other wide-field clinical images, increases usability as a triage tool by non-dermatologists. Any AI triage tools dependent on dermoscopy images, must assume that the end-user has professional training to identify lesions requiring specialist review. In the scenario of a telehealth TBP appointment where dermatologists are not involved in the imaging, the imaging technicians could be prompted by AI tools to take additional dermoscopy images of lesions flagged as suspicious or malignant.

A current challenge, particularly given the present melanoma overdiagnosis epidemic, is identifying those who are at the highest risk of melanoma and would benefit the most from screening and surveillance (50). Existing risk stratification methods often rely on subjective assessments or self-report which result in varying levels inter- and intra-rater reliability and can lead to misclassification (51, 52). This protocol uses experts to provide ground truth ratings using reproducible strategies, resulting in high quality data for training machine learning algorithms. Once trained, the algorithms will be able to provide automated and objective measures of phenotypic risk factors, removing the subjectivity of single raters and self-report.

Previously, the use of conventional total body photography imaging combined with sequential digital dermoscopy imaging has been shown to reduce the number of biopsies taken, as well as increasing the accuracy and timeliness of diagnosis in people at high risk of melanoma (53, 54). Therefore, incorporating an objective lesion-based risk as well as personal-based risk with these technologies has the potential to further aid in the early detection of melanoma, and reduce biopsies of benign lesions.

## Conclusion

This protocol describes the methods to construct a comprehensive dataset for machine learning, encompassing sequential-TBP, lesion classification, phenotype information, and patient-level metadata. The outcome of this study is to introduce a holistic approach for AI-driven CAD tools for the early detection of melanoma. This protocol addresses limitations of clinical transferability of existing algorithms, by including contextual clinical information in training datasets that are normally considered by a dermatologist. The incorporation of these tools with the Intelligent Total Body Scanner (iToBoS), will produce an innovative platform to improve patient risk stratification, and surveillance for early signs of skin cancer.

## Data availability statement

The original contributions presented in the study are included in the article/supplementary material, further inquiries can be directed to the corresponding author.

## Ethics statement

The studies involving humans were approved by the University of Queensland HREC (2022/HE001866) and Hospital Clinic of Barcelona HREC (HCB/2022/1051). The studies were conducted in accordance with the local legislation and institutional requirements. The participants provided their written informed consent to participate in this study.

## Author contributions

CP: Conceptualization, Data curation, Investigation, Methodology, Writing – original draft. BB-S: Conceptualization, Methodology, Writing – review & editing. NA: Conceptualization, Funding acquisition, Methodology, Writing – review & editing. BD: Conceptualization, Formal analysis, Methodology, Writing – review & editing. SG: Conceptualization, Data curation, Funding acquisition, Methodology, Writing – review & editing. PF: Conceptualization, Funding acquisition, Methodology, Writing – review & editing. AG: Conceptualization, Data curation, Formal analysis, Funding acquisition, Investigation, Methodology, Writing – review & editing. SG-V: Conceptualization, Data curation, Formal analysis, Funding acquisition, Investigation, Methodology, Writing – review & editing. KL: Conceptualization, Methodology, Writing – review & editing. SN: Conceptualization, Funding acquisition, Investigation, Methodology, Writing – review & editing. HN: Conceptualization, Funding acquisition, Investigation, Methodology, Writing – review & editing. VN: Conceptualization, Data curation, Formal analysis, Funding acquisition, Investigation, Methodology, Writing – review & editing. FP: Conceptualization, Data curation, Formal analysis, Funding acquisition, Investigation, Methodology, Writing – review & editing. BP: Conceptualization, Data curation, Formal analysis, Funding acquisition, Methodology, Writing – review & editing. JQ: Conceptualization, Data curation, Formal analysis, Funding acquisition, Investigation, Methodology, Writing – review & editing. SP: Conceptualization, Investigation, Methodology, Writing – review & editing. GR: Conceptualization, Investigation, Methodology, Writing – review & editing. RG: Conceptualization, Funding acquisition, Methodology, Project administration, Writing – review & editing. HS: Conceptualization, Funding acquisition, Methodology, Project administration, Writing – review & editing. JM: Conceptualization, Data curation, Funding acquisition, Methodology, Project administration, Writing – review & editing.
